# Gender-specific effects of transthyretin on neural stem cell fate in the subventricular zone of the adult mouse

**DOI:** 10.1038/s41598-019-56156-w

**Published:** 2019-12-23

**Authors:** Pieter Vancamp, Jean-David Gothié, Cristina Luongo, Anthony Sébillot, Karine Le Blay, Lucile Butruille, Maurice Pagnin, Samantha J. Richardson, Barbara A. Demeneix, Sylvie Remaud

**Affiliations:** 1 Muséum National d’Histoire Naturelle, CNRS UMR 7221, F-75005 Paris, France; 2 Department of Neurology & Neurosurgery, Montreal Neurological Institute & hospital, McGill University, Montreal, QC H3A 2B4 Canada; 3 School of Health & Biomedical Sciences, RMIT University, Bundoora, Victoria 3083 Australia; 4 School of Science, RMIT University, Bundoora, Victoria 3083 Australia

**Keywords:** Neural stem cells, Gliogenesis, Glial stem cells, Neural stem cells

## Abstract

Choroid plexus epithelial cells produce and secrete transthyretin (TTR). TTR binds and distributes thyroid hormone (TH) to brain cells *via* the cerebrospinal fluid. The adult murine subventricular zone (SVZ) is in close proximity to the choroid plexus. In the SVZ, TH determines neural stem cell (NSC) fate towards a neuronal or a glial cell. We investigated whether the loss of TTR also disrupted NSC fate choice. Our results show a decreased neurogenic *versus* oligodendrogenic balance in the lateroventral SVZ of *Ttr* knockout mice. This balance was also decreased in the dorsal SVZ, but only in *Ttr* knockout male mice, concomitant with an increased oligodendrocyte precursor density in the corpus callosum. Quantitative RTqPCR analysis following FACS-dissected SVZs, or marked-coupled microbeads sorting of *in vitro* neurospheres, showed elevated *Ttr* mRNA levels in neuronal cells, as compared to uncommitted precursor and glial cells. However, TTR protein was undetectable *in vivo* using immunostaining, and this despite the presence of *Ttr* mRNA-expressing SVZ cells. Altogether, our data demonstrate that TTR is an important factor in SVZ neuro- and oligodendrogenesis. They also reveal important gender-specific differences and spatial heterogeneity, providing new avenues for stimulating endogenous repair in neurodegenerative diseases.

## Introduction

Thyroid hormones (THs) are vital for optimal functioning of various organs including the brain, heart and liver. The thyroid gland mainly secretes the prohormone 3,5,3′,5′-tetraiodothyronine (T_4_ or thyroxine) that reaches peripheral target tissues *via* the circulatory system, from whence it gets locally activated into the bioactive 3,5,3′-triiodothyronine (T_3_) by deiodinase enzymes. In mammals, the distributor proteins thyroxine-binding globulin, transthyretin (TTR) and albumin bind the lipophilic THs in the blood, thus counteracting partitioning into cell membranes^1,2^. In the central nervous system, TTR is produced by choroid plexus epithelial cells facilitating TH distribution across the blood-cerebrospinal fluid (CSF) barrier, allowing THs to reach various regions within the brain, one of the most TH-sensitive organs^3,4^.

The subventricular zone (SVZ), lining the lateral ventricular walls, is a key neurogenic region in close contact with the CSF^5^. This stem cell niche sustains lifelong *de novo* generation of neurons and oligodendrocytes^6^. Furthermore, T_3_ is a crucial signal determining SVZ-derived neural stem cell (NSC) fate, i.e. whether NSCs generate neuronal or oligodendroglial precursor cells (NPCs and OPCs, respectively). In mice, increased T_3_ levels interacting with TH receptor α1 (TRα1) stimulate NSCs to commit to the neuronal lineage, while TRα1 absence combined with high expression of the T_3_-inactivating deiodinase type 3 (DIO3) favours oligodendroglial commitment^7,8^. SVZ-derived OPCs differentiate into myelinating oligodendrocytes and were able to restore myelin thickness and nerve conduction in the surrounding white matter (e.g. corpus callosum) of mice following a demyelinating insult^8^.

As an effective TH distributor in the CSF, TTR could consequently be a key component affecting the neuro- and gliogenic capacities within the SVZ niche. *Ttr* knockout (KO) mice are viable and fertile^9^, but as expected have reduced T_4_ (52%) and T_3_ (86%) levels in the CSF^10^. Despite the absence of gross abnormalities in adult brain morphology^11^, apoptosis of post-mitotic cells in the adult SVZ was reduced^12^ together with observed proliferative defects^13^. Recent research has also attributed other roles to TTR that might transcend its well-known TH distribution function. *Do novo* TTR synthesis was observed in several neuronal populations in the murine cortex, striatum and cerebellum^14,15^, as well as in motor neurons and Schwann cells in the spinal cord^16–19^. While an intracellular role is yet to be defined, it was shown that TTR stimulates neuritogenesis in some neuronal cell types in a ligand-independent manner^19–21^.

Recent studies additionally indicated that SVZ generates distinct neuronal^22,23^ and oligodendroglial^24,25^ populations in a region-dependent manner. Moreover, several external cues can trigger a more pro-neurogenic or pro-oligodendrogenic state in these spatial microdomains^25–27^. In addition, a large-scale single cell RNA-seq analysis uncovered gender-related differences in OPC numbers in the septal and lateral SVZ walls^28^. This SVZ regionalization and gender dimorphism imply that the regulation of NSC activity is even more sophisticated than previously assumed, however the underlying molecular mechanisms remain poorly explored. So far, there is scarce evidence indicating if and how TH action contributes to gender dimorphism in the neuro- and oligodendrogenic potential of the SVZ. This is particularly relevant to neurodegenerative diseases, which are characterised by neuronal or glial cell loss, and show gender-specific susceptibility. For instance, two to three-times more women than men are affected by multiple sclerosis (MS), the most common demyelinating disorder^29^. It has been shown that TH is required for efficient remyelination^30,31^.

Here, we used *Ttr* KO mice to further study the contribution of TTR in NSC fate in the adult mouse SVZ. We report that TTR absence differentially affects neuron/glia balance in the SVZ microdomains. By combining *in vitro* and *in vivo* approaches, we also found *Ttr* mRNA expression in SVZ cells committed to the neuronal lineage, but despite the presence of *Ttr* mRNA-expressing cells *in vivo*, the protein itself was undetectable. Moreover, we observed gender dimorphism within microdomains, opening new paths of exploration to understand how TH physiology contributes to sex-specific phenotypes. These findings could have important consequences for understanding NSC biology and help to highlight the role of TTR in pathological contexts, such as MS.

## Results

### *Ttr* knockout mice display gender-independent increased oligodendrogenesis in the lateroventral SVZ

**Figure 1:**
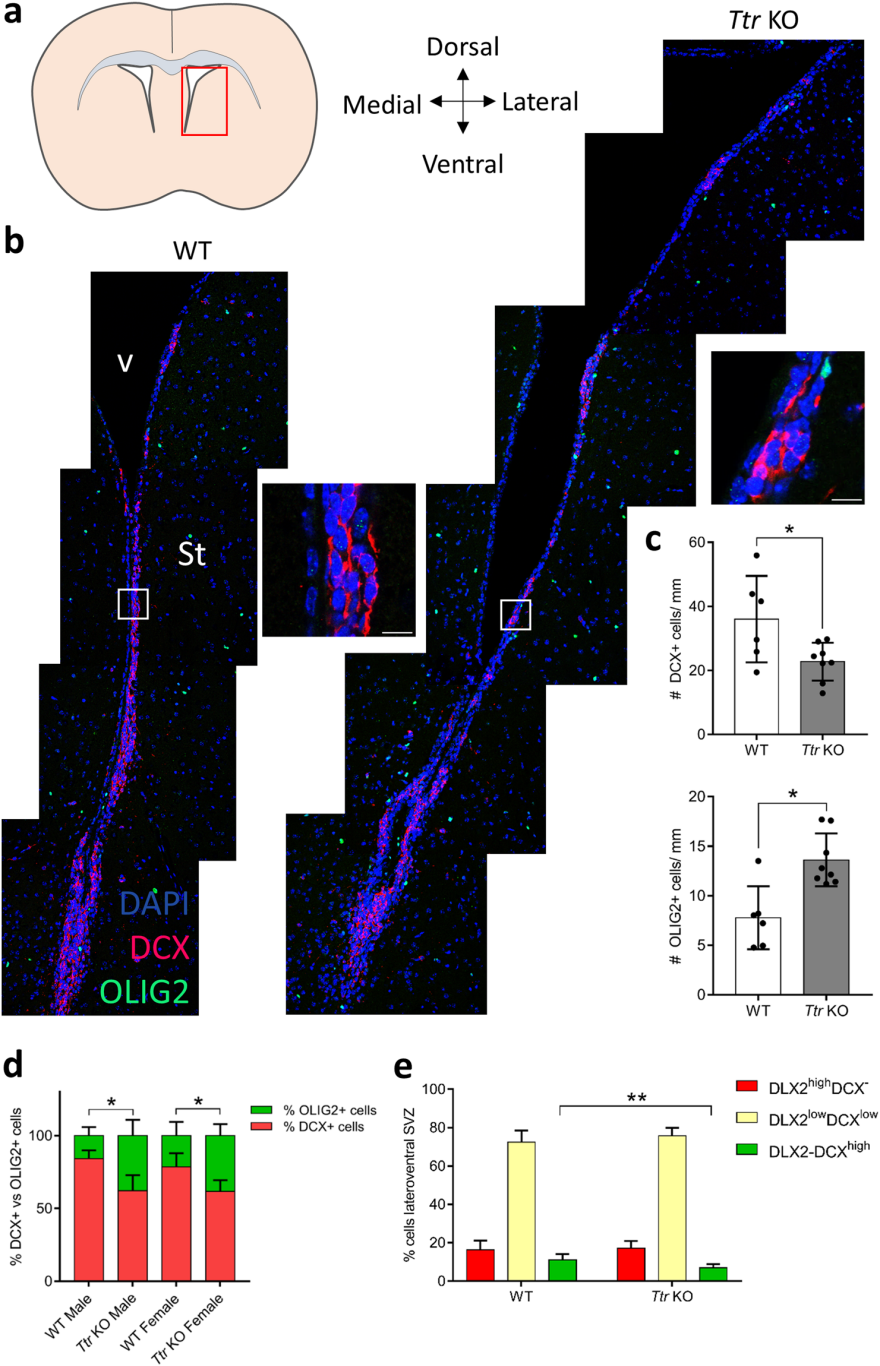
*Ttr* KO results in decreased neuroblast and increased OPC generation in the lateroventral SVZ. (**a**) Schematic representation of the region of interest (the lateroventral SVZ, red box). (**b**) DCX+ neuroblasts (red) and OLIG2+ OPCs (green) are observed across the dorso-ventral axis of the lateroventral SVZ. Inserts show DCX+ and OLIG2+ cells in detail (white boxes). Scale bars: 10 µm. (**c**) Statistical analysis revealed a significant decrease in DCX+ cells in *Ttr* KO mice as compared to WTs, contrasting a significant increase in OLIG2+ cells (n = 6–8, two-tailed Student’s T test). (**d**) The ratio of DCX+ neuroblasts *vs*. OLIG2+ OPCs in the lateroventral SVZ is reduced in both male and female *Ttr* KO mice as compared to WTs (n = 3–4, one-way ANOVA, followed by Tukey Post-hoc test). (**e**) *Ttr* KO mice display a significant reduced number of DLX2^−^DCX^high^-expressing mature neuroblasts (green bars), suggesting hampered neuronal differentiation (n = 6–8, two-tailed Student’s T tests). Bars represent mean ± SD. St: striatum; v: ventricle. *P < 0.05, **P < 0.01.

We assessed whether TTR regulates neuro- and oligodendrogenesis in the SVZ of adult mice by analysing consequences for the neuron/glia balance in the absence of TTR. Therefore, we used age-matched, wild-type (WT) and *Ttr* KO mice^12^ to study NSC fate determination *in vivo*. First, we quantified the number of both doublecortin (DCX)+ neuroblasts and OLIG2+ OPCs in the lateroventral SVZ by performing a co-immunostaining on coronal brain slices (Fig. 1a,b). In *Ttr* KO mice, the number of DCX+ neuroblasts was 37% lower as compared to WT mice (T test, t_12_ = 2.514, P = 0.027), while the number of OLIG2+ OPCs was 39% higher (Mann-Whitney U test, P = 0.013) (Fig. 1c). This indicates that TTR absence results in a shift towards oligodendrogenesis at the expense of neurogenesis. Furthermore, comparison of the proportion of DCX+ over OLIG2+ cells showed that this effect was similar in males (T test, t_5_ = 3.174, P = 0.025) and females (T test, t_5_ = 2.622, P = 0.047) (Fig. 1d).

Second, we analysed whether early neuronal commitment was also impaired in *Ttr* KO mice. We performed a co-immunostaining for the early NPC marker Distal-Less Homeobox 2 (DLX2) and the mature neuroblast marker DCX on coronal brain slices. To determine young uncommitted and committed *versus* mature neuronal populations, three groups were examined: NPCs (DLX2^high^DCX^−^), young committed neuroblasts (DLX2^low^DCX^low^), and mature neuroblasts (DLX2^−^DCX^high^). While the proportion of both DLX2^high^ uncommitted NPCs and DLX2^low^DCX^low^ young differentiating neuroblasts in the lateroventral SVZ were similar (DLX2^high^: T test, t_12_ = 0.34, P = 0.74; DLX2^low^DCX^low:^ T test, t_12_ = 1.237, P = 0.24), the proportion of DCX^high^ mature neuroblasts was approximately 1.5-fold lower in *Ttr* KO mice as compared to WT mice (T test, t_12_ = 3.315, P = 0.0061) (Fig. 1e). These results indicate that neuronal differentiation is hampered or delayed in the lateroventral SVZ microdomain in absence of TTR.

### *Ttr* knockout mice display decreased neurogenesis in the dorsal SVZ in a gender-specific manner

**Figure 2:**
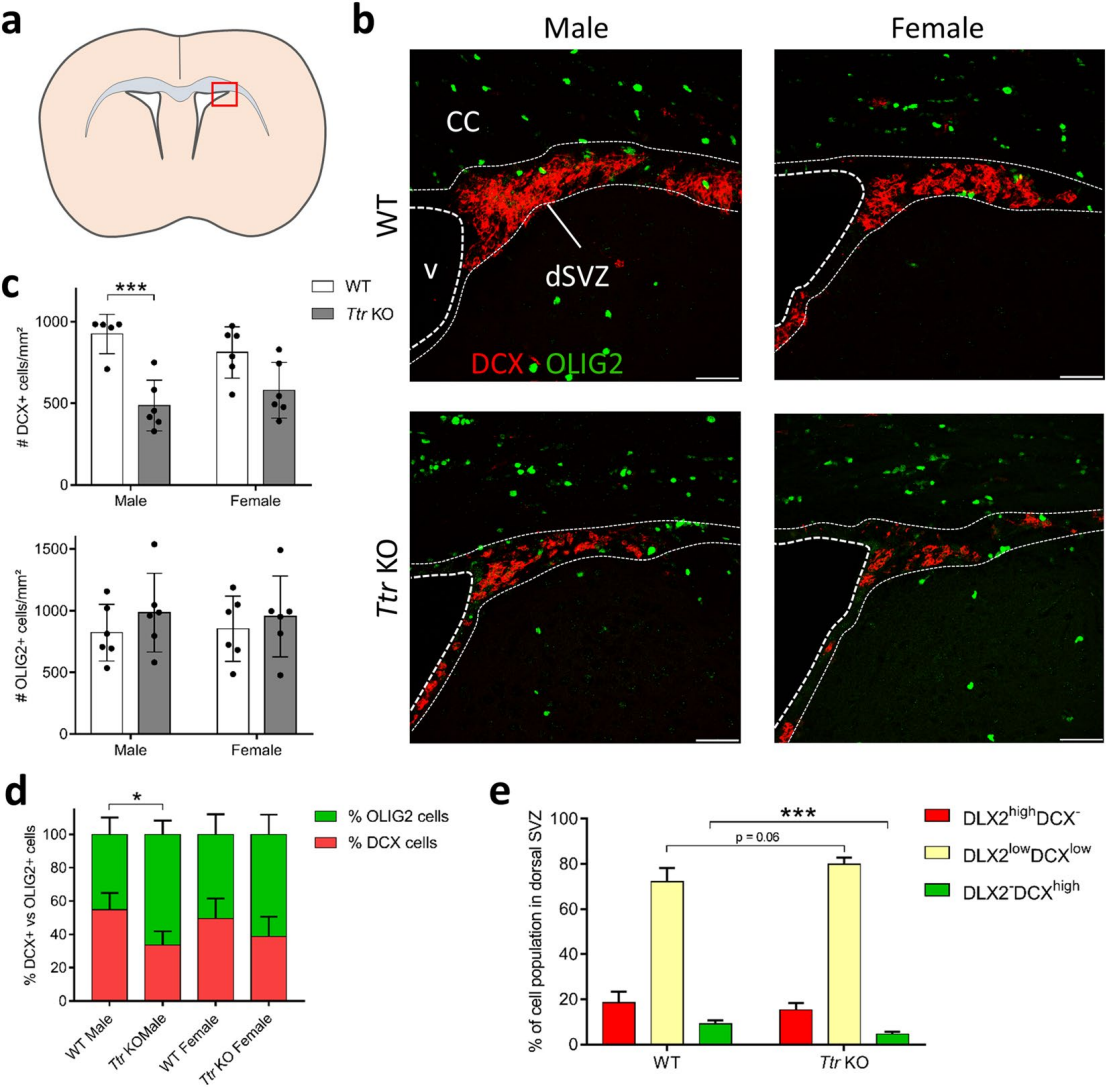
*Ttr* KO results in decreased neuroblast numbers in the male dorsal SVZ. (**a**) Schematic representation of the region of interest (the dorsal SVZ, red box). (**b**) DCX+ neuroblasts (red) densely populate the dorsal SVZ in males and females, together with several OLIG2+ OPCs (green). The DCX+ cell density in *Ttr* KO mice is clearly lower, while OPC numbers are similar. Scale bars: 20 µm. (**c**) Statistical analysis revealed a significantly reduced DCX+ cell number in the dorsal SVZ of male *Ttr* KO mice as compared to WTs (n = 5–6, two-way ANOVA, followed by Tukey Post-hoc test). (**d**) The ratio of DCX+ neuroblasts *versus* OLIG2+ OPCs in the dorsal SVZ is reduced in males only (n = 5–6, two-way ANOVA, followed by Tukey Post-hoc test). (**e**) *Ttr* KO mice display a significant reduced number of DLX2^−^DCX^high^-expressing mature neuroblasts, and a borderline-significant increase in DLX2^low^DCX^low^-expressing immature neuroblasts, suggesting hampered neuronal differentiation (n = 6–8, two-tailed Student’s T tests). Bars represent mean ± SD. CC: corpus callosum; dSVZ: dorsal subventricular zone; v: ventricle. *P < 0.05, ***P < 0.001.

Next, we performed the DCX/OLIG2 co-immunostaining to investigate the effect on neuro- and oligodendrogenesis in the dorsal SVZ (Fig. 2a,b). The number of DCX+ neuroblasts per area (mm²) was significantly reduced, nearly 2-fold in *Ttr* KO male mice only, as compared to WT mice (Tukey Post-hoc following two-way ANOVA, q_19_ = 6.691, P = 0.00077). In contrast, OLIG2+ OPC numbers were not affected in either *Ttr* KO male or female mice (Tukey Post-hoc following two-way ANOVA, F_(1,20)_ = 1.261, P = 0.275) (Fig. 2c). Since the absence of TTR affected neurogenesis in a gender-specific way, we calculated the proportion of DCX+ *versus* OLIG2+ cells to assess NSC fate choice in both genders. We observed a significant genotype-effect (two-way ANOVA, F_(1,19)_ = 12.72, P = 0.0021) (Fig. 2d). Post-hoc analysis showed that the neuron/glia ratio shifted from approximately 55%/45% to 30%/70% in male *Ttr* KO mice (Tukey Post-hoc, q_19_ = 4.639, P = 0.019), while being unaffected in females (q_19_ = 2.443, P = 0.34). Altogether, these results indicate that TTR absence decreased neurogenesis, thus promoting SVZ-oligodendrogenesis in the dorsal SVZ of male mice only.

We also quantified the percentage of DLX2^high^DCX^−^ uncommitted NPCs, DLX2^low^DCX^low^ young committed neuroblasts and DLX2^−^DCX^high^ mature neuroblasts and observed a similar effect on neuronal lineage progression in the dorsal SVZ, as previously seen in the lateroventral SVZ. A borderline-significant increase in young committed neuroblasts (T test, t_12_ = 2.056, P = 0.062) and a significant reduction of mature neuroblasts (T test, t_12_ = 4.453, P < 0.001) was observed in *Ttr* KO mice compared to WT mice. NPC numbers were unchanged (T test, t_12_ = 0.2441, P = 0.81), suggesting that early neuronal fate determination was not affected (Fig. 2e). Again, these data suggest hampered neuronal differentiation, resulting in a 2-fold reduction of mature neuroblasts in the dorsal SVZ due to the absence of TTR in both genders.

### Proliferation and cell cycle progression of neural progenitors are not affected in *Ttr* knockout mice

**Figure 3:**
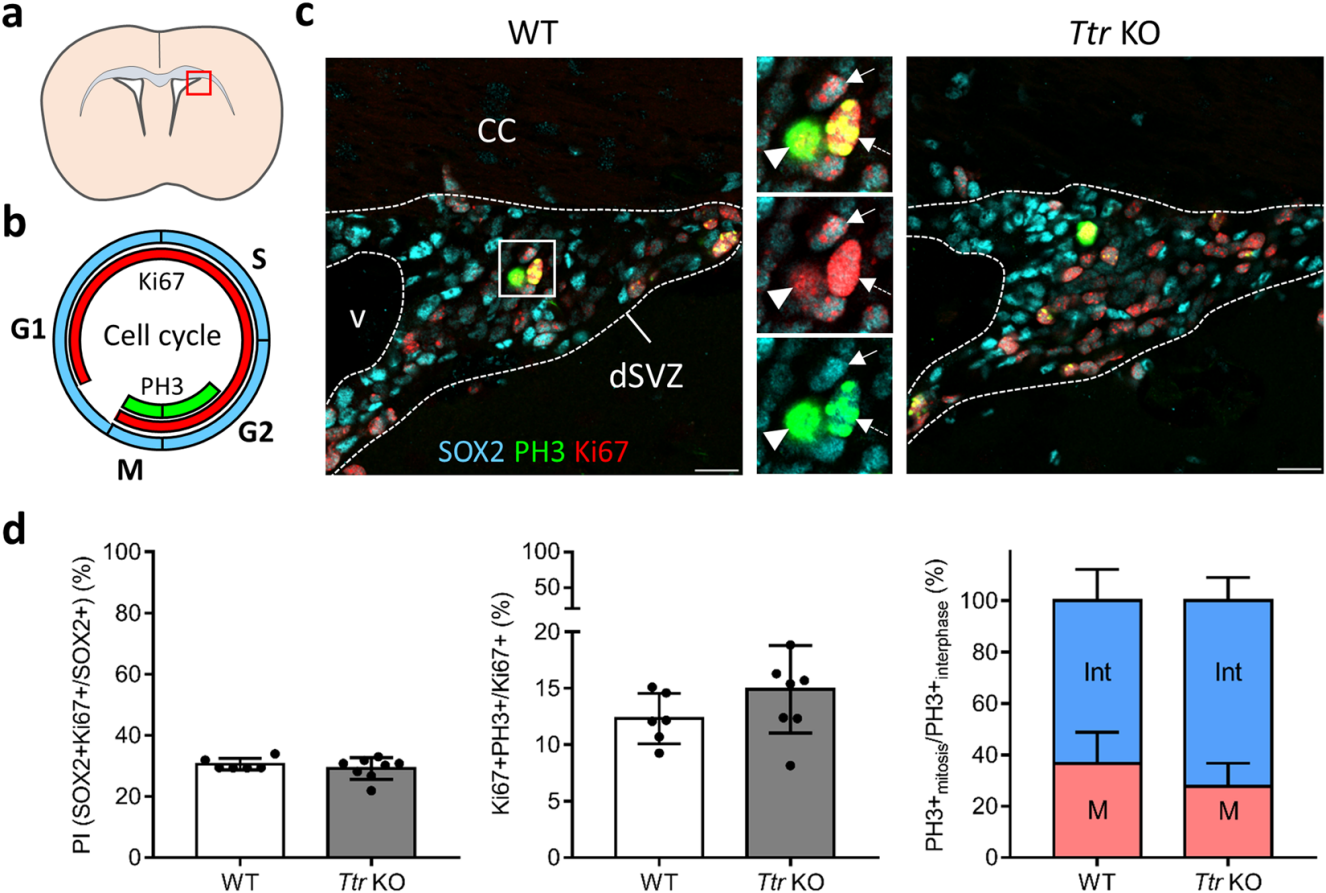
*Ttr* KO does not affect progenitor cell proliferation and cell cycle progression. (**a**) Schematic representation of the SVZ region highlighted in C (the dorsal SVZ, red box). (**b**) Scheme of the cell cycle, divided in four phases: S, G2, M, and G1. Ki67 (red) is expressed throughout the almost entire cell cycle, while PH3 (green) marks the late interphase (G2) and mitosis (M). (**c**) Representative pictures of a WT and *Ttr* KO dorsal SVZ showing SOX2+ progenitors (light blue), Ki67 (red), and PH3 (green). The inserts show some SOX2+ cells expressing Ki67 (arrow). PH3+ cells have a dotted staining pattern in late G2-phase (dotted arrow), and a pan-nuclear staining pattern during the M-phase (arrowhead). No obvious changes were noticeable in *Ttr* KO mice. Scale bars: 20 µm (**d**) The proliferation index (left graph), the proportion of Ki67+ cells co-stained with PH3 (middle graph), as well as the ratio of PH3+ cells in G2- and M-phases (right graph) are similar between WT and *Ttr* KO mice (n = 6–8, two-tailed Student’s T tests or Mann-Whitney U test). Bars represent mean ± SD. CC: corpus callosum; dSVZ: dorsal subventricular zone; Int: interphase; M: mitosis; PI: proliferation index; v: ventricle.

We tested whether the significant reduction in mature neuroblasts in *Ttr* KO mice could be due to a lower proliferative activity of neural progenitors expressing SOX2, a gatekeeper of NSC identity (Fig. 3a). We therefore performed a triple immunostaining against SOX2 combined with two well-established markers of cell proliferation: Ki67, marking all cell cycle phases, and phospho-histone 3 (PH3), a marker for the late G2- and M-phases (Fig. 3b). We quantified the number of SOX2+ NSCs/progenitors expressing Ki67 alone (cycling cells) or both Ki67 and PH3 (G2- and M-phase cells) in the dorsal (Fig. 3c) and lateroventral SVZ (not shown). The average percentage of Ki67+ cells in the SOX2+ population (e.g. the proliferative index (PI)) is approximately 30% in the dorsal SVZ of both WT and *Ttr* KO mice (Mann-Whitney U test, P = 0.76) (Fig. 3d: left graph). Furthermore, around 14% of the dividing cells co-expressed Ki67 and PH3 in both WT and *Ttr* KO mice (T test, t_12_ = 1.466, P = 0.168) (Fig. 3d: middle graph). Finally, we assessed cell cycle progression by calculating the proportion of PH3+ cells in mitosis (pan-nuclear staining) *versus* interphase (dotted-staining pattern) and found no significant differences between both groups (T test, t_12_ = 1.567, P = 0.14) (Fig. 3d: right graph). All above analyses were additionally performed on the lateroventral SVZ of WT and *Ttr* KO mice, whereby statistical significant differences were not achieved in the PI (T test, t_12_ = 0.2976, P = 0.77), or proportion of Ki67+ PH3 co-expressing cells (T test, t_12_ = 0.795, P = 0.44) and the proportion of PH3+ cells in G2- *versus* M-phase (Mann-Whitney U test, P = 0.414). Altogether, these results strongly indicate that absence of TTR neither affects the proliferative activity, nor cell cycle progression of adult SVZ-progenitor cells.

### Male *Ttr* knockout mice display an increased OPC density in the corpus callosum

**Figure 4:**
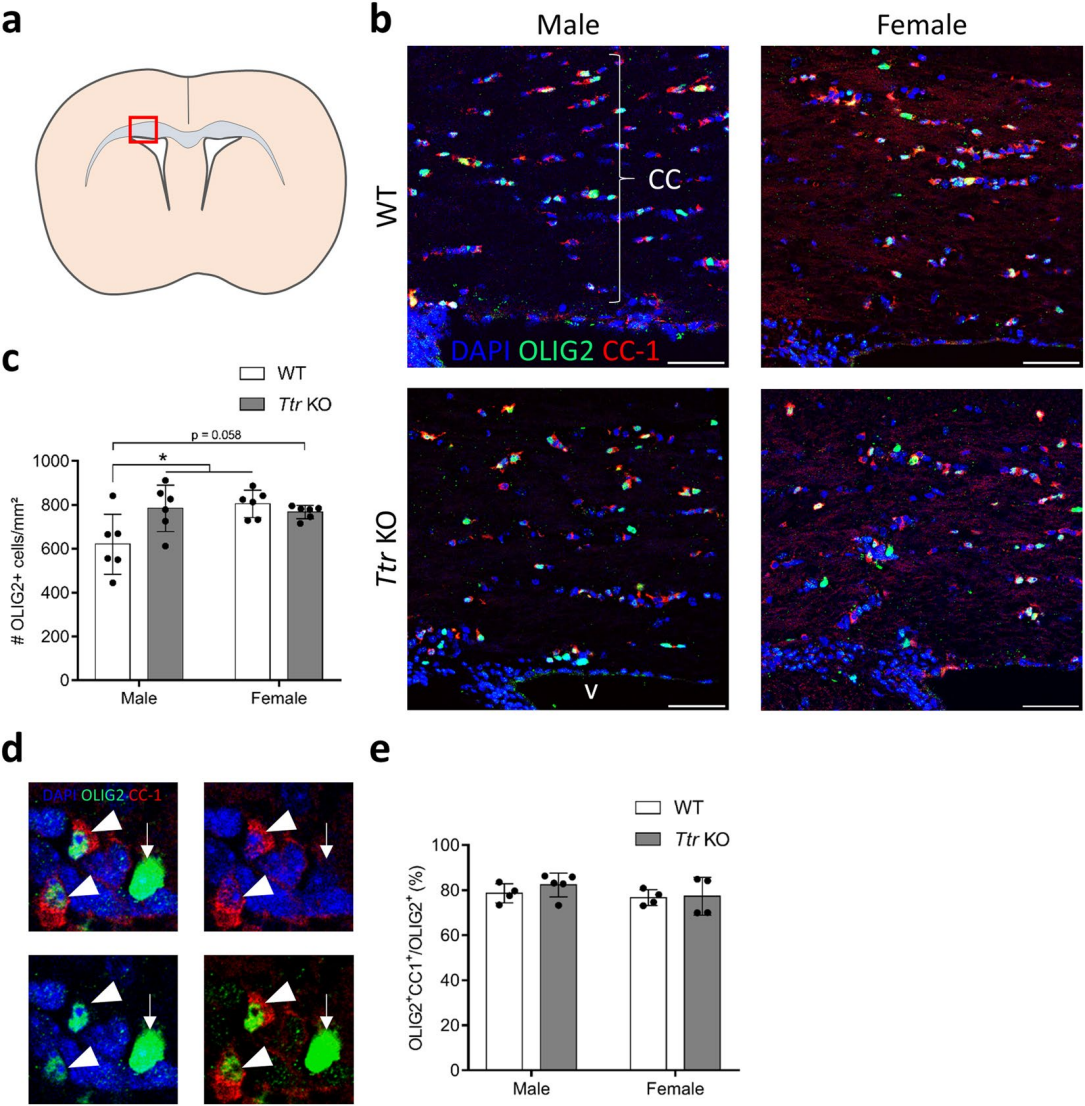
*Ttr* KO results in increased pOPC numbers in the corpus callosum of male mice only. (**a**) Schematic representation of the region of interest (corpus callosum, red box). (**b**) OLIG2+ oligodendroglial cells (green) are abundantly present in the corpus callosum of WTs. The majority of them are mature oligodendrocytes also expressing CC-1 (red). Scale bars: 50 µm. (**c**) Statistical analysis revealed that OLIG2+ cell number per mm² in WTs is higher in females, and that *Ttr* KO results in a significantly increased pOPC number in males only, reaching WT female values (n = 6, two-way ANOVA, followed by Tukey Post-hoc test). (**d**) Detailed pictures showing mature oligodendrocytes co-expressing OLIG2 (green) in the nucleus and CC-1 (red) in the cytoplasm (arrowheads), while immature pOPCs only express OLIG2 (arrow). (**e**) The proportion of OLIG2+ cells co-expressing CC-1 does not differ statistically between all groups (n = 4–5, two-way ANOVA). Bars represent mean ± SD. CC: corpus callosum; v: ventricle. *P < 0.05.

We previously demonstrated that resident parenchymal OPCs (pOPCs) located in the corpus callosum do not respond to a transient lack of TH, contrary to SVZ-OPCs^8^. These resident pOPCs are generated during development and persist during adulthood, in contrast with the newly generated SVZ-OPCs^32^. Therefore, we examined whether TTR absence also affected the density of all oligodendrocyte lineage cells expressing OLIG2 (OLIG2+ pOPCs) in WT and *Ttr* KO mice, in both genders (Fig. 4a,b). We found a significant gender effect (two-way ANOVA, F_(1,20)_ = 4.804, P = 0.040) and an interaction effect (F_(1,20)_ = 26.997, P = 0.016). The pOPC density was 21% higher in male *Ttr* KO mice as compared to WT males (Tukey Post-hoc, q_20_ = 4.306, P = 0.030), while this effect was absent in female mice (q_20_ = 0.985, P = 0.90) (Fig. 4c). This finding shows that the oligodendroglial population within the male corpus callosum is more sensitive to a lack of TTR than that of females. Interestingly, the pOPC density in female WT mice was significantly higher than in male WTs (Tukey post-hoc, q_20_ = 4.837, P = 0.013). Furthermore, male *Ttr* KO mice only reached female WT pOPC numbers in a TTR-absent environment (Fig. 4c).

Next, we assessed whether the lack of TTR affects oligodendroglia subpopulations at varying maturity within the corpus callosum. To this end, we immunostained the protein adenomatous polyposis coli (APC, also known as CC-1) that marks mature, myelinating oligodendrocytes. This enabled us to calculate the ratio of OLIG2+ CC1 co-expressing mature oligodendrocytes *versus* immature pOPCs expressing OLIG2 alone (Fig. 4d). On average 80% of the OLIG2+ cells in the corpus callosum co-expressed CC-1, and no significant differences were found between WT and *Ttr* KO mice (two-way ANOVA, F_(1,13)_ = 0.610, P = 0.45), neither in males, nor in females (F_(1,13)_ = 1.591, P = 0.23) (Fig. 4e). This suggests that only immature pOPCs numbers in the corpus callosum differ between genders and genotypes, whereas TTR does not affect the generation of mature oligodendrocytes.

### Intracellular *Ttr* mRNA is differentially expressed amongst SVZ cell types

**Figure 5:**
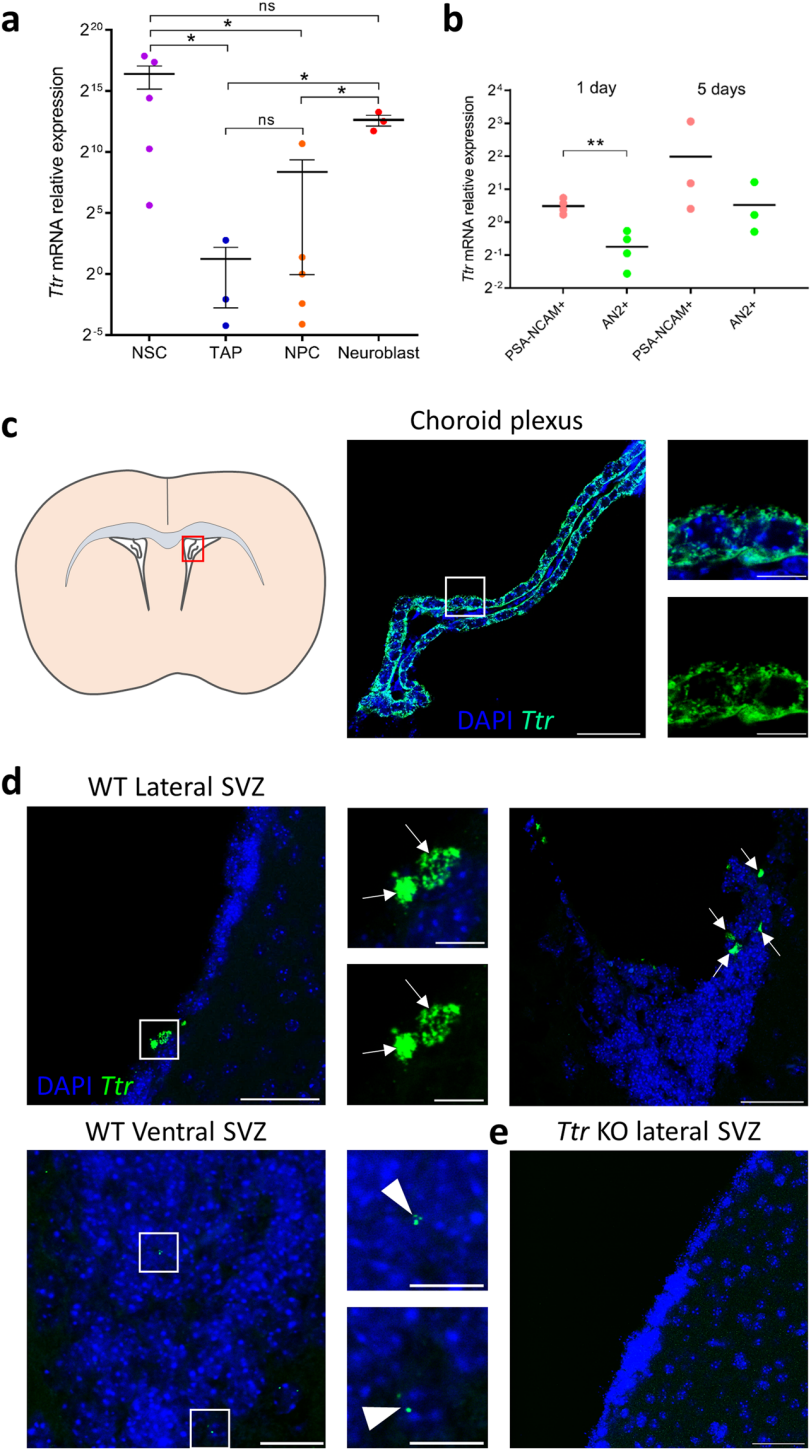
Detection of *Ttr* mRNA expression in SVZ cells. (**a**) *Ttr* mRNA expression levels in FACS-sorted SVZ cells. High *Ttr* levels were found in quiescent and activated NSCs (CD133+), after which they dropped in TAPs (EGFR+) and NPCs (EGFR+ CD24+). Cells committed to the neuroblast lineage (CD24+) showed increased *Ttr* expression levels (n = 3–5, Kruskal Wallis ANOVA followed by Mann-Whitney U tests). (**b**) *Ttr* mRNA expression levels were measured in magnetic bead-sorted neuronal (PSA-NCAM+) and oligodendroglial (AN2+) cells after 1 and 5 days of *in vitro* differentiation. *Ttr* mRNA levels were significantly higher in neuronal cells after 1 day of *in vitro* differentiation (n = 3–4, two-tailed Student’s T tests). (**c**) Visualisation of *Ttr* mRNA transcripts in choroid plexus epithelial cells (red box on the schematic section) using RNAscope. *Ttr* mRNA transcripts (green dots, magnification of the white box) are abundantly present in the cytoplasm surrounding the nuclei (blue). Scale bar left picture: 50 µm, inserts: 10 µm. (**d**) Representative pictures of the lateral and ventral SVZ of WT mice (n = 4) showing few cells with a high number of *Ttr* mRNA transcripts (white arrows, magnification of white boxe), and some cells with a few transcripts (arrowheads, magnification of white boxes). Scale bar overview pictures: 50 µm, inserts: 10 µm. (**e**) No transcripts were detected in *Ttr* KO mice (n = 3). Scatter dot plots represent mean ± SEM. NPC: neural precursor cell; ns: not significant; NSC: neural stem cell; TAP: transient amplifying progenitor. *P < 0.05. **P < 0.01.

Since *Ttr* mRNA and protein have been detected in several neuronal cell types throughout the nervous system, we determined whether SVZ-derived cells also express *Ttr*. Therefore, we first examined *Ttr* expression at the transcriptional level after separating SVZ cell types using fluorescence-activated cell sorting (FACS). We purified NSCs for positive expression of the marker CD133^33^ (activated NSC, aNSC) or negative to CD133 expression (quiescent NSC, qNSC) with EGFR^34^. Transit amplifying cells (TAPs) (EGFR+ CD133−CD24−) and mature neuroblasts (EGFR−CD24+) were separated using a combination of antibodies against EFGR and CD24^35^. Quantitative PCR analysis revealed that *Ttr* expression significantly changed during neuronal differentiation (Kruskal Wallis ANOVA, P = 0.018), starting with a strong decrease during the cell transition from NSC to TAP (Mann-Whitney U test, P = 0.025), followed by an increase during the last step of neuronal commitment (Mann-Whitney U test, P = 0.049) (Fig. 5a). These results indicate that *Ttr* mRNA is expressed in SVZ-derived cells, especially during neuronal cell fate determination.

Then, we further assessed *Ttr* expression in neuronal *versus* oligodendroglial cells by generating neurospheres from dissected adult SVZs, which were cultured in the presence of growth factors for 7 days. Neurospheres were dissociated, plated and allowed to differentiate for 1 or 5 days. Neuroblasts and OPCs were sorted at both time points using magnetically labelled microbeads coupled with antibodies directed against Polysialylated-neural cell adhesion molecule (PSA-NCAM) and the proteoglycan AN2, respectively. We studied gene expression by RTqPCR in both cell types, at early differentiation (1 day, 1d) and late differentiation (5 days, 5d). At 1d, *Ttr* expression was significantly higher in PSA-NCAM+ neuroblasts compared to AN2+ OPCs (T test, t_6_ = 5.355, P = 0.0017), while no significant difference between lineages was detected at 5d (T test, t_6_ = 1.133, P = 0.32) (Fig. 5b).

### *Ttr* mRNA, but not protein was detected in the SVZ *in vivo*

We used the RNAscope assay to visualise *Ttr* transcripts in the SVZ. The assay was accompanied by the appropriate positive and negative control probes, as recommended by the manufacturer (see Materials & Methods). We performed our assay using the *Ttr* probe on coronal brain sections of postnatal day 4 (P4) mice and validated the abundant presence of *Ttr* mRNA transcripts in the cytoplasm of choroid plexus epithelial cells, as expected (Fig. 5c). The assay was simultaneously performed on coronal sections through the SVZ of WT and *Ttr* KO adult mice of both genders (n ≥ 3 per group) (Fig. 5d,e). Only a few cells in the lateral and ventral SVZ expressed high amounts of *Ttr* mRNA transcripts, visible as punctate dots, each representing one mRNA transcript. Some cells expressed a low amount of *Ttr*, between 1 and 5 transcripts per cell (Fig. 5d). Surprisingly, the majority of the SVZ cells *in vivo* did not express a single *Ttr* transcript. Moreover, the dorsal SVZ was completely absent of signal.

**Figure 6:**
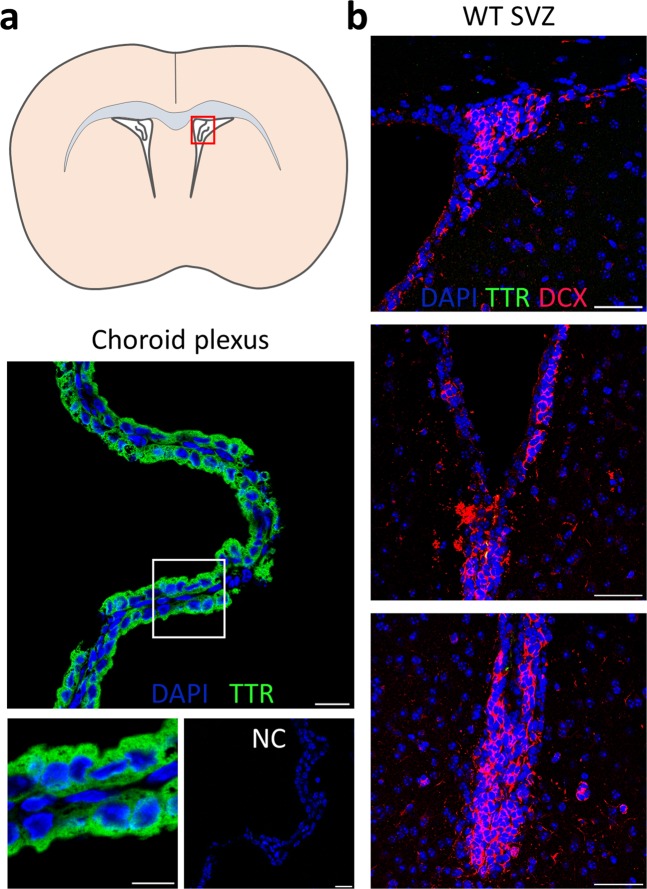
TTR protein is undetectable in the SVZ *in vivo*. (**a**) TTR staining using the monoclonal Rabbit-anti-mouse TTR antibody on coronal sections from the P4 WT brain shows strong specific signal in the choroid plexus (red box), as expected. Inserts (magnification of white box) show TTR presence in the cellular cytoplasm, while no TTR is detected in the negative control. Scale bars: 50 µm, insert: 10 µm. (**b**) No TTR signal was detectable in the dorsal, lateral or ventral SVZ of coronal brain sections of neither male, nor male adult WT mice (n ≥ 3 per group). Scale bars: 50 µm. NC: negative control.

The changing *Ttr* mRNA expression pattern prompted us to investigate whether TTR protein was also present in SVZ cells *in vivo*, ultimately to evaluate whether it contributed to the observed phenotype. Therefore, we used two distinct rabbit antibodies to visualise TTR: one monoclonal Rabbit-anti-mouse TTR antibody (Abcam, ab215202) against the full-length protein, and one polyclonal Rabbit-anti-human TTR (Abbiotec, ab250892) against a sequence within the C-terminus region, each with the appropriate positive and negative controls. Furthermore, we amplified the signal using the TSA Fluorescein System (Perkin Elmer, see Material and Methods section). We observed a strong positive TTR signal in the choroid plexus of P4 mice with both antibodies, as expected, while the negative controls (absence of the primary antibody in WT animals, and sections of *Ttr* KO mice (data not shown for the latter)) did not display specific staining. Figure 6a shows the staining with the Rabbit-anti-mouse TTR antibody that gave the clearest results. However, no TTR staining was detected on coronal brain sections of male and female WT mice used for the phenotypical analysis, neither in the dorsal, nor in the lateroventral SVZ (Fig. 6b). This may indicate that *Ttr* mRNA is not translated into TTR protein, or that TTR levels are too low to be detected by immunohistochemistry in SVZ cells *in vivo*.

## Discussion

TTR production and secretion by choroid plexus epithelial cells facilitates the distribution of THs *via* the CSF, enabling access to target brain cells^1,3,4^. SVZ-NSCs line the lateral ventricular walls and have apical processes that are in direct contact with the CSF^5,36^, and thus are potential key target cells for TTR-mediated TH supply. Moreover, T_3_ and its receptor TRα1 are crucial for determining NSC fate choice in the adult murine SVZ^7,8,37^. *Ttr* KO mice display reduced cellular apoptosis in the adult murine SVZ^12^ but evidence for a role in neuro- and oligodendrogenesis was missing, prompting us to investigate how the absence of TTR affected NSC fate. Our work shows that TTR differentially affected NSC fate in distinct SVZ microdomains and uncovers an unexpected gender effect in the regulation of the neuro *versus* oligodendroglia balance.

### TTR absence increases oligodendrogenesis in the SVZ, probably due to local hypothyroidism

We found an almost 40% increase in OPC generation in the lateroventral SVZ of *Ttr* KO mice, together with a diminished neuroblast generation. In contrast, OPC numbers remained stable in the dorsal SVZ, whereas neuroblast numbers decreased. These results indicate that NSC fate choice in the SVZ is partially regulated by TTR. Since CSF T_4_ and T_3_ levels of *Ttr* KO mice are reduced by 52 and 86%, respectively^10^, our *in vivo* observations corroborate the hypothesis that TTR absence results in a reduced TH availability in SVZ cells^12^. Correspondingly, a transient period of hypothyroidism also changed the neuro/glia balance towards a pro-oligodendrogenic state in the adult murine SVZ^8,37^. Palha and coworkers showed that T_4_ levels and deiodinase type 2 activity in the cortex, cerebellum and hippocampus of *Ttr* KO mice were unchanged, as were whole brain parenchyma T_3_ levels and the expression of the TH-responsive gene *RC3*^10^, which can explain the absence of gross brain abnormalities^11^. This suggests that, in contrast to other brain regions, the SVZ (which is in close proximity to the choroid plexus) is a special niche that is particularly sensitive to changes in CSF-mediated supply of THs and other factors. Accordingly, a transcriptomic analysis demonstrated that SVZ-NSC dynamics are highly sensitive to a multitude of choroid plexus-derived signals^5^.

We also found that neuronal differentiation, a typically TH-sensitive process corresponding to the transition from NPCs to mature neuroblasts^38^, was hampered, as shown by significantly decreased mature neuroblast numbers combined with a slight accumulation of young committed NPCs. Elevated NPC numbers in *Ttr* KO mice could therefore also be caused by dysregulated processes other than blocked migration^13^. However, the proliferative activity as well as the cell cycle progression of neural progenitors were not affected throughout the entire SVZ of *Ttr* KO mice. This is in line with earlier studies showing similar numbers of cycling Ki67-positive and mitotic PH3-positive cells^12^ plus BrdU-positive cells^39^ in the SVZ of WT and *Ttr* KO mice. Only one study found increased PCNA (a S-phase marker) labelling in the *Ttr* KO SVZ, suggesting enhanced cell proliferation^13^. These findings differ from observations in hypothyroid mice, which displayed increased BrdU-positive cell numbers but a strong reduction in Ki67- and PH3-positive cells, indicating a block in cell cycle progression^40^. It is also unlikely that cell apoptosis contributes to the observed effects, since the absence of TTR led to a 2-fold decrease of the few apoptotic cells that were detected in the entire SVZ of WT mice^12^.

Other regulators, such as TH transporters and deiodinases enable cells to regulate their intracellular T_3_ concentrations tightly^41^ thus allowing T_3_-target cells to modify their cell fate in the adult SVZ. Our group already demonstrated that intracellular T_3_ depletion by Deiodinase type 3 and the combined absence of the TH receptor α1 modulate NSC fate preferentially toward SVZ-OPCs^8^, whereas a transitory expression of TRα1 drives SVZ-NSCs toward a neuronal fate^7^. It would therefore be of interest to investigate NSC fate in mice deficient in TH transporters (MCT8, OATP1C1) and/or deiodinases (DIO2, DIO3) too. The interaction of all these TH signalling components ultimately defines how cellular SVZ responses are regulated following injury.

### Gender dimorphism in *Ttr* KO mice suggests different SVZ-OPC generation capacities

The corpus callosum is a highly myelinated region involved in inter-hemisphere transmission of sensory information^42^. In the adult, it contains resident pOPCs, originating from a pool of embryonic NSCs, as well as newly generated OPCs by the pro-oligodendrogenic dorsal SVZ domain^8,25,43^. Trace mapping showed that newly generated SVZ-derived OPCs can efficiently migrate and populate the corpus callosum, participating in remyelination following a demyelination insult in the adult^8,43^, while resident pOPCs hardly responded to a transient hypothyroid state^8^. Here, we also found increased OPC numbers in the corpus callosum, located dorsal of the lateral ventricle of male *Ttr* KO mice. The relatively high OPC density in the dorsal SVZ compared to the lateroventral SVZ (compare Figs. 1d with 2d) suggests that this microdomain, in accordance with previous studies^44^, could thus be an important OPC source, especially in situations of demyelinating damage^8,43^. We did not find any differences in the proportion of mature oligodendrocytes in all groups, suggesting that only newly generated SVZ-OPCs are affected by the absence of TTR.

In females, OPC numbers in the corpus callosum were not significantly altered in the absence of TTR. In addition, we did not observe any gender-specific differences in the neuro/glia ratio in the lateroventral SVZ, suggesting that SVZ-oligodendrogenesis could be distinct along the entire SVZ between males and females. Similar gender-related differences were observed in other studies as well. White matter volume in the corpus callosum was higher in male than in female rats^45^. Thirty to 40% more mature oligodendrocytes were counted in the corpus callosum of WT male mice as compared to females^46^. A recent single cell RNA-seq analysis also identified spatial heterogeneity and sex differences in OPC populations within the lateral and septal adult SVZ. In this study, the lateral wall was more pro-neurogenic as compared to the pro-oligodendrogenic septal wall, with a strong OPC enrichment in the latter, in males only^28^. In addition, exposure to polychlorinated biphenyl during development and the resulting hypothyroxinaemia, increased expression of myelin basic protein (*MBP*) (a marker of mature oligodendrocytes) levels in male mice but led to the opposite effect in females^47^. This suggests that TTR-dependent TH supply provokes gender-specific disparities linked to regional heterogeneity in the regulation of SVZ-NSC fate.

Gender effects and SVZ heterogeneity can provide clues to help explain the shift in MS prevalence from 2:1 to 3:1 in women *versus* men and the variable disease progression between both sexes^29,48,49^. Following a demyelinating insult, more efficient remyelination was also observed in old female rats compared to their male counterparts^50^. Recently, TH analogues have gained interest as potential therapeutic agents combatting demyelinating diseases such as MS, by specifically stimulating the endogenous repair potential^25,27,51^. However, oestrogen cycles in females could interact with the action of the TH analogues, impeding reliable outcomes in short-term experiments^51^. Similarly, TH administration in the acute phase of demyelination rescued normal MBP expression and myelin sheath assembly^52^. However, only female rats were used, questioning to what extent this TH treatment may have beneficial effects on male rats. Hence, our data and that of others suggest analysing effects of THs and thyromimetics on remyelination should be carried out as a function of gender.

### Presence of mRNA, but undetectable TTR protein most likely excludes an intracellular role in SVZ cells

Lastly, we assessed whether *Ttr* mRNA is expressed *de novo* in SVZ-derived cells. We found high *Ttr* mRNA expression in NSCs, which were strongly reduced in TAPs, and increased again in committed NPCs and mature neuroblasts. In contrast, glial cells did not express detectable levels of *Ttr*. Marker-conjugated microbeads sorting of neuronal and glial cell types confirmed that only cells committed to the neuronal lineage express *Ttr*.

A microarray analysis on neurospheres from dissected mouse adult SVZs identified *Ttr* as one of the most highly enriched genes in the SVZ^53^. However, using the RNAscope assay, we observed a high amount of *Ttr* mRNA transcripts only in a few cells within the lateroventral SVZ. The majority of SVZ-cells expressed either a few transcripts or nothing at all. The identity of isolated cells expressing high *versus* low levels of *Ttr* mRNA remains to be elucidated.

Remarkably, no TTR protein was detected in the male, nor the female SVZ *in vivo* using two commercially available antibodies. We can either hypothesize (i) that translation into protein is very low to absent, (ii) that there is a high turnover, (iii) that TTR is rapidly secreted or (iv) that it serves no function in SVZ cells. Alternatively, it could be that *de novo Ttr* mRNA expression starts in NPCs and will later be translated in neuroblasts migrating along the rostral migratory stream or in differentiating interneurons in the olfactory bulb. In fact, recent data provides evidence that both embryonic and adult mammalian NSCs transcribe high amounts of mRNA but post-translational repression mechanisms postpone protein production until neuronal differentiation starts^54,55^. Intracellular TTR protein production has been observed in other differentiated cell types of the murine nervous system, for instance in motor neurons^19^ and Schwann cells^18^, although its function is still unclear. TTR can also be internalised by at least some neurons such as hippocampal neurons, where it subsequently acts as a ligand-independent, neurotrophic, and neuroprotective factor following cerebral ischemia, by stimulating neuritogenesis and neuronal recovery through the MAPK pathway^56^. In another experiment, TTR administration obstructed *in vitro* neurosphere expansion and inhibited cell proliferation. In this case, it might function as a sequestering protein for the pro-mitotic, bioactive T_3_^57^. One could speculate that *Ttr* mRNA expression and translation into functional protein is a default state in some neurons that are farther away from the CSF, while NSCs in close contact to the CSF are supplied with sufficient TTR, therefore post-translationally block protein generation.

Some unresolved issues require further investigation. For instance, TTR also forms a complex with retinol-binding protein, delivering retinol (vitamin A) throughout the circulation and CSF^58^. Retinol and its active metabolite retinoic acid have been shown to increase the generation of SVZ-neuroblasts in the murine postnatal brain^59^. Therefore, we cannot exclude that some effects could be partly due to reduced retinol delivery. Another remaining question is to what extent the observed effects are permanent consequences of TTR absence during development, rather than solely attributed to effects in the adult SVZ. *Ttr* KO mice display disrupted brain development, during which resident pOPC populations are established in the developing white matter^11^. Moreover, NSCs probably undergo important epigenetic changes during development, which at least partially program their neuro- and gliogenic capacity for the rest of life^6,60^. However, the SVZ retains its neuro- and oligodendrogenic capacity and sensitivity to internal and external cues throughout life. That could enable the SVZ to respond to pathophysiological insults or damage, even though the self-repair capacity is very limited, especially in mammals. This could explain why only the SVZ seems to be affected by TTR absence in adult mice, while other brain regions are not influenced^12,13,39^, possibly due their reliance on other TH supply mechanisms such as blood brain-barrier mediated TH uptake.

## Materials and Methods

### Animals

C57BL/6J WT male mice, 8 weeks old, were purchased from Janvier (Le Genest St. Isle, France), and WT and *Ttr* KO male and female mice were obtained from a specialised facility at the RMIT University Animal Facility (RMIT University, Victoria, Australia) for *in vivo* studies. They were kept in ventilated cages in a 12/12 h dark-light regimen, provided with *ad libitum* water and food (rat chow containing 0.5 mg/kg iodine).

All procedures were conducted according to the principles and procedures in Guidelines for Care and Use of Laboratory Animals and validated by local and national ethical committees (Australian National Health and Medical Research Council guidelines, approval by the RMIT University Animal Ethics Committee (AEC# 1209)).

### Immunohistochemistry

Mice were anesthetized with Pentobarbital (130 mg/kg, Centravet) and perfused rapidly through the left heart ventricle with 1X PBS, followed by 4% paraformaldehyde (PFA) in PBS. Brains were harvested and post-fixed at 4 °C overnight in the same fixative solution. Brains were cryoprotected in 30% sucrose in 1X PBS at 4 °C overnight, embedded in OCT (Sakura), and stored at −80 °C. Thirty µm thick coronal brain sections were made using a cryostat and were collected in cold, sterile 1X PBS. Sections were incubated for 1 h in a blocking solution (10% donkey serum (Sigma), 1% BSA (Sigma), 1X PBS with 0.05% Triton X-100) at room temperature (RT) and then incubated with primary antibodies diluted in blocking solution overnight at 4 °C. Following 3 × 10 min washes in 1X PBS at RT, sections were incubated with Alexa-conjugated, fluorescent secondary antibodies (Invitrogen) (1/500, 1% donkey serum, 1% BSA in 1X PBS) for 2 h at RT. The antibodies for TTR were detected using the TSA Fluorescein System (NEL701A001KT, Perkin Elmer). Sections were then washed 3 × 10 min in 1X PBS at RT, incubated with DAPI for 5 min at RT and mounted on coated, SuperFrost+ glass slides (Thermo Fisher Scientific) and covered with Prolong Gold containing antifade reagent (Invitrogen).

Antibodies and their dilution factors were as follows: Rabbit anti-TTR (1/200, Abbiotec), Rabbit anti-TTR (1/10000, Abcam), Goat anti-DCX (1/500, Santa Cruz); Guinea Pig anti-DCX (1/500, Millipore), Guinea Pig anti-DLX2 (1/3000, gift from K. Yoshikawa laboratory), Rabbit anti-NG2 (1/300, Millipore), Rabbit anti-OLIG2 (1/300, Millipore), Mouse anti-APC (1/300, Millipore), Mouse anti-PH3 (1/800, Millipore), Goat anti-SOX2 (1/200, Santa Cruz), Rabbit anti-Ki67 (1/400, Abcam).

### RNAscope

Visualisation of mRNA transcripts was performed using the commercially available RNAscope^®^ Multiplex Fluorescent Reagent Kit v2 Assay (Product Nr° 323100-USM, Advanced Cell Diagnostics, Hayward, CA, USA). The RNAscope^®^ Probe-Mm-*Ttr* (Cat No. 424171, Accession number: NM_013697.5) was used to detect *Ttr* mRNA transcripts. Two RNAscope^®^ positive controls, the Probe-Mm-*Ubc* and Probe-Mm-*Ppib*, were used to validate homogenous high and medium expression of the *Ubc* and *Ppib* gene, respectively. The RNAscope^®^ negative control Probe-Mm-*DapB* was used to check for background signal. Coronal sections of at least 3 mice per genotype of both genders were used. P4 brain sections containing choroid plexus served as an additional positive control for *Ttr* expression, while sections of *Ttr* KO mice were used as an additional negative control. The RNAscope experiment was repeated twice.

Free-floating sections were first mounted on SuperFrost+ glass slides (Thermo Fisher Scientific) and baked at 60 °C for 1 h to ensure adherence to the slides. The baking procedure was repeated once after dipping the slides 3 x in distilled H_2_O (dH_2_O) and afterwards sections were dipped in 100% Ethanol. The slides were then placed in mild-boiling (98–102 °C) Target Retrieval Reagent for 15 min followed by 3 washes in dH_2_O and 1 wash in fresh 100% ethanol. Slides were air-dried and baked at 60 °C for another 30 min. All following steps were performed in the HybEZ™ II Oven (Advanced Cell Diagnostics, Hayward, CA, USA) at 40 °C, and between every two steps all slides were washed 3 × 5 min in 200 mL Wash Buffer. First, five drops of Protease solution were applied on each section for 30 min. Then, 5 drops of the Probe were applied per section and incubated for 2 h. Next, sections were incubated for 30 min with 5 drops of RNAscope^®^ Multiplex FL v2 Amp1, 30 min with RNAscope^®^ Multiplex FL v2 Amp2, and 15 min with RNAscope^®^ Multiplex FL v2 Amp3. To develop the signal, 5 drops of RNAscope^®^ Multiplex FL v2 HRP-C1 were applied on each section for 15 min, followed by 150 µL of a fluorescent dye (Opal^TM^ 520 for the Probe-Mm-*Ttr* and Probe-Mm-*DapB*, Opal^TM^ 570 for the Probe-Mm-*Ubc*, and Opal^TM^ 690 for the Probe-Mm-*Ppib*) for 30 min, and lastly RNAscope^®^ Multiplex FL v2 HRP blocker for 15 min. This last cycle was repeated with RNAscope^®^ Multiplex FL v2 HRP-C2 when two signals were developed on the same section. Five drops of DAPI were applied for 90 s on each section at RT, prior to mounting with Prolong Gold containing antifade reagent (Invitrogen).

### FACS experiments

The lateral SVZs of five adult WT male mice were dissected and incubated at 37 °C in papain for 30 min with pipette dissociation every 10 min to obtain a single-cell suspension. After resuspension, cells were treated with a debris removal solution (Miltenyi). Cells were then incubated with a combination of the following antibodies: an Alexa488-conjugated Rat anti-mouse antibody directed against CD133 (Clone 13A4, Thermo Fisher Scientific), a BV421-conjugated Rat anti-mouse antibody against CD24 (BV421-CD24, BD Biosciences), and an APC-conjugated antibody against EGFR (E13345, Thermo Fisher Scientific) for 30 min at 4 °C. Cell sorting was performed on a BD FACSAria^TM^ III, using BD FACSCanto^TM^ II for the subsequent analysis. After 10 min centrifugation at 2000 rpm, cell pellets of CD133+ EGFR− qNSCs, CD133+ EGFR+ aNSCs, EGFR+ CD133−CD24− TAPs, EGFR+ CD24+ NPCs, and CD24+ EGFR− mature neuroblasts were frozen at −20 °C until RNA extraction.

### Neurosphere assays

Primary adult NSC neurosphere cultures were generated as previously described^37^. Briefly, the lateral SVZs of five adult WT male mice were dissected per neurosphere culture and incubated in papain solution for 30 min at 37 °C with pipette dissociation every 10 min to obtain a single-cell suspension. Cells were then cultured in proliferating conditions (with addition of growth factors EFG and FGF) for 7 days at 37 °C to obtain primary neurospheres. To analyse cell differentiation, neurospheres were dissociated into single cells and plated on Poly-D-Lysine-coated glass coverslips in 24-well plates (50000 cells per well) in complete culture medium without proliferating factors for 1 to 5 days.

For immunocytochemistry experiments, cells were fixed with 4% PFA for 10 min at RT. Cells fixed on glass coverslips were blocked with 10% donkey serum and 1% BSA (Sigma) in PBS for 30 min at RT, and incubation with primary antibodies was performed in blocking solution overnight at 4 °C. After 3 × 5 min washes in 1X PBS at RT, cells were incubated with Alexa-conjugated fluorescent secondary antibodies (Invitrogen) (1/500, in 1% donkey serum and 1% BSA in PBS) for 1 h at RT. Cells were washed 3 × 5 min in 1X PBS, incubated with DAPI for 5 min at RT and mounted on SuperFrost+ glass slides (Thermo Fisher Scientific).

### Microbeads cell sorting

After 24 h or 5 days differentiation *in vitro*, cells derived from neurosphere cultures were incubated at 37 °C with trypsin (Gibco) for 90 s, and gently detached by pipetting in inactivating culture medium containing 10% FBS. After resuspension, cells were treated with a debris removal solution (Miltenyi) and sorted using anti-AN2 MicroBeads (Miltenyi) following the manufacturer’s instructions. AN2- cells were then sorted with anti-PSA-NCAM MicroBeads (Miltenyi) to collect neuronal lineage cells. PSA-NCAM+ cells and AN2+ glial cells were centrifuged and pellets were frozen at −20 °C until RNA extraction.

### RNA extraction and quantitative PCR

RNA extraction and reverse-transcription were done using the RNAqueous-Micro kit with DNase treatment (Thermo Fisher Scientific) and the Reverse Transcription Master Mix (Fluidigm), following the manufacturer’s instructions. Pre-amplifications were performed for genes of interest using the Taqman Preamp Master Mix kit (Thermo Fisher Scientific), with primers from Taqman Gene expression assays: *Dcx*, Mm00438400_m1; *Dlx2*, Mm00438427_m1; *Gapdh*, Mm99999915_g1; *ActB*, Mm01205647_g1; *Cspg4* (NG2), Mm00507256_m1; *Sox10*, Mm00569909_m1; *Ttr*, Mm00443267_m1. Detection of the PCR products was monitored by measuring the increase in fluorescence generated by Taqman probes for every sample, in triplicates. ΔCt values were calculated by normalizing Ct with the mean of *ActB* and *Gapdh* housekeeping genes Ct values. To limit any experimental variation in the gene analysis of AN2+/PSA-NCAM+ cells, every ΔCt was normalized by the mean ΔCt of the AN2+ and PSA-NCAM+ samples (1d differentiation) from the same experiment (4 technical replicates per condition).

### Imaging

Fluorescent images were acquired using a Leica TCS-SP5 confocal microscope. All *in vitro* quantifications were done on Max Intensity Z projections of 4 µm stack images. All *in vivo* quantifications were done on 30 µm thick brain slices located between bregma +0.4 mm and +0.9 mm stereotaxic coordinates. Pictures were taken consistently on two sections per mouse, and the dorsal, lateral and ventral SVZ of both sides were imaged on each section. In addition, three detailed pictures were taken of the corpus callosum at each side to quantify the number of resident pOPCs and mature oligodendrocytes. RNAscope imaging was also performed on the confocal microscope. Cell counting was performed in the freely available FIJI software, which contains a cell counter plugin. Brightness and contrast were adapted for optimal counting, without losing any biological information.

### Statistical analysis

GraphPad Prism software 7.00 was used for all statistical analyses. Data were first checked for normality using the Kolmogorov-Smirnov test. Comparison of means between two groups was done using the two-tailed Student’s T test (parametric) or the Mann-Whitney U test (non-parametric). Comparison of a variable between more than two groups was done using a one-way ANOVA (parametric) or Kruskal-Wallis test (non-parametric), unless stated otherwise. When applicable, the ANOVA was followed by a Tukey Post-hoc test, and the Kruskal Wallis test by a Dunn’s Post-hoc test, to determine which means differed from one another. In cases where there were two independent variables, a two-way ANOVA was used followed by a Tukey Post-hoc test. For the comparison of relative *Ttr* mRNA expression of FACS-sorted cells, each pair of group means was tested separately by Mann-Whitney U tests following a Kruskal Wallis ANOVA, using StatXact 11 software. For all tests, α = 0.05 was chosen as level of significance. *In vitro* data are presented as scatter dot plots including mean and standard error of the mean (SEM), while *in vivo* data are presented as a combination of scatter dot plots and bars depicting mean and standard deviation (SD). P values and the corresponding statistical test are given throughout the text, while the levels of significance are represented on the graphs with asterisks: *P < 0.05, **P < 0.01 or ***P < 0.001.

## Data Availability

The datasets generated and/or analysed during the current study are available from the corresponding author on reasonable request.
